# Selenomethionine-Dominated Selenium-Enriched Peanut Protein Ameliorates Alcohol-Induced Liver Disease in Mice by Suppressing Oxidative Stress

**DOI:** 10.3390/foods10122979

**Published:** 2021-12-03

**Authors:** Lin Gao, Jiawei Yuan, Yuhuan Cheng, Mengling Chen, Genhua Zhang, Jihong Wu

**Affiliations:** 1 School of Biology and Food Engineering, Changshu Institute of Technology, Changshu 215500, China; gaolin2017@cslg.edu.cn (L.G.); yuanjiawei5008@163.com (J.Y.); Chengyuhuan0625@163.com (Y.C.); cml19801003@163.com (M.C.); zgh@cslg.edu.cn (G.Z.); 2 National Engineering Research Center for Fruits and Vegetables Processing, Ministry of Science and Technology, Beijing 100083, China; 3 College of Food Science and Nutritional Engineering, China Agricultural University, Beijing 100083, China

**Keywords:** selenium, alcohol-induced liver disease (ALD), selenomethionine (SeMet), oxidative stress, *Nrf-2*

## Abstract

Numerous natural compounds are considered as potential therapeutic agents against alcohol-induced liver disease (ALD). Research shows that selenium (Se) has a variety of bioactivities, including liver protecting ability. The present study based on in vitro cell culture models and in vivo mouse models was aimed at examining the contribution of selenomethionine (SeMet)-dominated Se-enriched peanut protein (SePP) to liver protection. SeMet and especially SePP reversed cell viability and cell death, inhibited ethanol induced CYP2E1 activation, decreased reactive oxygen species level, and restored GSH level. Hence, SeMet-dominated SePP alleviates alcohol-induced AML-12 cytotoxicity by suppressing oxidative stress. The *p38*-dependent mechanism was found to be responsible for SePP-induced *Nrf-2* activation. Furthermore, supplementation with SePP and SeMet regulated lipid metabolism and reduced oxidative stress, minimizing liver damage in mice. Selenomethionine-dominated SePP possesses potential therapeutic properties and can be used to treat ALD through the suppression of oxidative stress.

## 1. Introduction

The prevalence of liver disease has considerably increased worldwide [1]. Liver disease caused by alcohol, also called alcoholic liver disease (ALD), is one of leading external causes of mortality [2]. Various diseases are proven to be associated with oxidative stress, which refers to the negative effects of free radicals in the body [3]. Studies show that ALD development can be exacerbated by reactive oxygen species (ROS) formation and antioxidant activity reduction [2,4]. In recent decades, plant-based materials, plants, or their active extracts have been determined as potential therapeutic agents of interest for the prevention and treatment of ALD, owing to their low toxicity and multi-target effects [5,6]. For example, pine nut (*Pinus koraiensis)* polysaccharides and ginsenosides in *ginseng* wine have a protective effect on alcohol-induced liver damage [7,8,9].

Selenium (Se), an essential trace element, has attracted scientific attention for its beneficial effects against antioxidant activity-related diseases, such as cancers, cardiovascular and liver diseases. Chemoprevention trials mostly utilize commercially available reagents, including sodium selenite, methylseleninic acid (MSeA), selenomethionine (SeMet) and selenocysteine (SeCys) [10]. Evidence suggests that organic Se compounds are safer and better absorbed and utilized than inorganic Se. Notably, in foods organic species of Se are obtained through the conversion of inorganic Se in the soil to selenoproteins by plants [11]. Reportedly, MSeA can significantly enhance apoptosis in breast cancer cells [12]. SeCys is an important component of glutathione peroxidase 4 [13], which is required to prevent Ferroptosis by suppressing oxidative stress. Among several Se compounds, SeMet is important owing to its non-specific incorporation into proteins [14] and is most easily absorbed by the human body, as the bioavailability of different Se species ranks as SeMet (56%) > SeMeCys (46%) > Se(VI) (33%) > Se (IV) (12%) [15]. SeMet plays an important role in accelerating cell viability and growth [16]. Moreover, Se is principally supplemented as SeMet as it is the predominant Se species found naturally in foods, such as cereals, rice, beans, Se yeast, and nuts. For instance, 5.75 to 6.26 mg/kg SeMet was found in walnuts [17], and 21.77 mg/kg SeMet was identified in Brazil nuts [11].

Research shows the effects of Se treatment on the chemical compositions and antioxidant properties of some plant extracts [18,19]. There are also some relevant studies on Se and liver health. The liver and kidneys are considered to be the organs with the highest capacity to accumulate Se [20,21]. Moreover, low Se intake is associated with a high risk of liver diseases. Se can promote the repair and regeneration of liver cells by supplementing trace elements in patients with hepatitis, fatty liver or cirrhosis [22]. Se-containing proteins and low-molecular Se compounds can effectively inhibit hepatocarcinogenesis in transgenic mice [23]. Reportedly, the damage and necrosis of liver cells in Se-deficient patients are similar to those caused by excessive alcohol consumption [24].

Agronomic biofortification through soil application is a cost-effective method to increase Se concentration in edible portions of crops [15]. Peanuts (*Arachis hypogeae*) are rich in proteins, accounting for ~25% of the dry weight of seeds and, thus, are a high-quality material for food processing and an ideal carrier for Se biofortification [15,25]. Our earlier study reveals that the dominant Se species in Se-enriched peanuts is SeMet, accounting for 65% of the total Se content in peanuts [26]. Herein, we extended our study to gain Se-enriched peanut protein (SePP) and explored their possible activity in liver protection through both in vitro cell culture models and in vivo mouse models. Then, the possible hepatoprotective mechanisms were investigated by assessing the oxidative stress. Our study is highly innovative as it is the first to focus on the effects of Se-enriched peanut extract and its dominating Se compound on liver protection. The findings will provide new insights into ALD prevention based on plant-sourced Se.

## 2. Materials and Methods

### 2.1. Peanut Protein Preparation

Se-enriched peanuts and common peanuts (*Arachis hypogeae*) were provided by the Ecological Selenium Valley Modern Agriculture Management Committee (Fengcheng, China). Sodium selenite was added to the soils in which the Se-enriched peanuts were grown. Peanut proteins were prepared according to Gao et al. [26]. In brief, the peanut oil was removed with high hydrostatic pressure of 300 MPa for 5 min and heated in a water bath (37 °C, 150 rpm/min) for 12 h twice. Then, peanut proteins were extracted as follows: 50 °C, pH 9.0, 95 min, liquid-to-material ratio 11 mL/g, and precipitation pH 5.5. The extract as obtained was then freeze-dried.

Physical and chemical indexes in the SePP powder were detected according to the National Standards of China (http://www.sac.gov.cn/, accessed on 13 March 2021). For instance, protein content, crude fiber, crude fat, moisture and ash were monitored according to GB 5009.5-2016, GB/T 5009.10-2003, GB/T 5009.6-2016, GB 5009.3-2016, and GB 5009.4-2016, respectively.

### 2.2. Detection of Total Selenium and SeMet Contents

SeMet standards (seleno-D, L-methionine 99%), SeCys_2_ (seleno-L-cystine 95%), MeSeCys (Se-methylselenocysteine 95%), and sodium selenite (99%) were purchased from Sigma Aldrich Co. (Shanghai, China). Standard solutions of Se, Ge and Kr were produced by the National Institute of Metrology (Beijing, China). All other chemicals and reagents used here were of analytical grade and bought from Sinopharm Chemical Reagent Co. Ltd. (Nanjing, China). Total Se content was detected by an inductively coupled plasma mass spectrometer (ICP-MS, PerkinElmer NexION 350X, Shelton, USA) [26]. At each time, a sample (about 0.5000 g) was weighed and put into a modified polytetrafluoroethylene digestion vessel. Then, 4 mL of nitric acid and 4 mL of hydrogen peroxide (30%, *v*/*v*) were added, and the resulting solution was digested with a microwave system using the program: 150 °C, 15 min; 170 °C, 15 min; 180 °C, 30 min. After digestion, the final volume was adjusted to 50 mL. A digestion blank was also applied. Moreover, 4% nitric acid solutions with Se standard solutions (1, 2, 5, 10, 20, 50 ppb) and 1 ppm internal standard solution (Ge, Rh) were prepared. Then, ^78^Se, ^72^Ge and ^83^Kr were monitored. Each sample was tested in triplicate.

SeMet content was detected using high-performance liquid chromatography (HPLC) -ICP-MS according to the method of Hu et al. [26]. Samples were prepared with protease and lipase (2:1, *w*/*w*) dissolved in Na_2_S_2_O_3_ (5 mmol/L) in 30 mmol/L Tris-HCl buffer, and ultrasonically assisted for 1 h combined with a water bath at 37 °C for 20 h. The liquid supernatant was collected and filtered consecutively through 0.45-µm nylon membranes. This process was repeated twice. The two supernatants were then analyzed by HPLC–ICP-MS. To separate Se compounds, an anion-exchange column (Hamilton RPR-X100) was used, and the mobile phase flow rate was 1 mL/min at 24 °C with 40 mM (NH_4_)_2_HPO_4_ (pH 6.0). For quantification, calibration standards of Se compounds were prepared from 1.0 to 100.0 μg/L. The correlation coefficient *R*^2^ was greater than 0.999, and the recovery rates ranged from 85% to 102%.

### 2.3. Cell Culture and Treatments

AML-12 (alpha mouse liver 12) cells obtained from the Cell Bank of Chinese Academy of Sciences were cultured according to the manufacturer’s manual. The culture medium was composed of DMEM/F12 + 10% FBS + 10 µg/mL Insulin + 5.5 µg/mL Transferrin + 40 ng/mL Dexamethasone + 1% P/S. After laying for 24–48 h, the medium was changed before starting the treatment with SePP or SeMet when the cells were 50–60% confluent.

### 2.4. Cell Viability and Cell Death

Cell growth and viability were tested by a cell counting kit-8 (CCK8) (Beyotime) according to the instructions. In general procedure, cells were cultured in a 96-well plate for 24 h, then subjected to alcohol at a certain concentration and cultured for another 24 h. To determine the optimal concentration of alcohol, different concentrations (0.1, 0.2, 0.5, 0.8, and 1.0 mmol/L) were used to induce cell injury. Then, the cells were treated with a certain concentration of 10 μM SePP or SeMet for 24 h. According to the preliminary experiment and instructions, the cells were seeded into 96-well plates at 5 × 10^3^ cells per well with a group of blank control wells and a group of untreated control wells. Each incubation was performed in six separate culture wells. After resin monomer treatment, 10 μL of CCK-8 solution was added to 100 μL of the culture medium and incubated for 2 h at 37 °C. Finally, the absorbance at 450 nm was measured.

Cell death was assessed using an Annexin V-FITC apoptosis detection kit (Beyotime, China) according to the manufacturer’s protocol. In a general procedure, the cells were washed, centrifuged, resuspended with a binding buffer, combined with Annexin V-FITC (5 μL), and then added with PI (5 μL). After incubation in the dark for 15 min, the mixture was added to a flow cytometer (Becton, Dickinson and Company, New York, NY, USA) for detection.

### 2.5. ROS Detection

ROS was measured using an ROS assay kit (Beyotime) according to the instructions [27]. Briefly, the cells were incubated with 10 mM 2,7-dichlorodi-hydrofluorescein diacetate (DCFH-DA) at 37 °C in the dark for 30 min, and then washed with a serum-free medium to fully remove the DCFH-DA that did not enter the cells. Then, ROS content was measured using the flow cytometer.

### 2.6. Western Blotting

The cell suspension was diluted with PBS (pH 7.2–7.4) until the cell concentration reached about 1 million/mL. Through repeated freezing and thawing or addition with tissue protein extraction reagents, the cells were destroyed to release the intracellular components. After centrifugation (3000 rpm, 15 min), the supernatant was collected. Western blotting was performed according to the method of Song et al. [28]. Briefly, samples were loaded onto a sodium dodecyl sulfate polyacrylamide gel electrophoresis (SDS-PAGE) system (Beyotime) and transferred to polyvinylidene fluoride (PVDF) membranes electrophoretically (Millipore, Billerica, MA, USA). The membranes were blocked with 5% non-fat milk in Tris-buffered saline and Tween 20 for 1 h at room temperature. The membranes were then rinsed three times consecutively with a TBST buffer, and then incubated for 1 h with 1:1000 of primary monoclonal antibodies: *β-actin*, *Nrf-2*, *HO-1*, *GCLC*, *p38*, *keap-1*, *JNK1/2* and *ERK1/2* (Cell Signaling Technology, Shanghai, China) in a TBST buffer containing 1% skim milk. After that, secondary antibodies reacted with the bolts at room temperature for 2 h before visualization and analysis by densitometric scanning (Image Quant TL7.0, GE Healthcare Bio-Sciences AB, Mississauga, ON, Canada). Band density was quantified using ImageJ (National Institute of Health, Bethesda, MD, USA).

### 2.7. Animals and Treatments

An eight-week experimental animal program in accordance with the National Guidelines for Experimental Animal Welfare was authorized by Experimental Animal Welfare Ethics Committee, Changshu Institute of Technology on 10 May 2020, with the project identification code of CSLG-2020-FL-36. Four-week-old male ICR mice (*n* = 36, Beijing Weitong Lihua Company, Beijing, China) with four animals per cage were maintained and provided with standard diet (H10010, Huafu Kang Biotechnology Co., Ltd., Beijing, China), which can provide 3.85 kcal/g diet, which is 70% of energy as carbohydrates (67.30 g/100 g), 20% as proteins (19.20 g/100 g), and 10% as fats (4.30 g/100 g). The mice were acclimatized to the new environment for one week, and then randomly assigned to four groups. Specifically, a normal control group (N, *n* = 9) was offered standard chow pellets, and an ALD group (ALD, *n* = 9) received ethanol (30%, *v*/*v*) by gavage (10 mL/kg bw/day). Moreover, a SePP group was offered a diet containing 2.575 g/kg bw/day SePP (25 μg/kg bw/day in Se), and a SeMet group was offered a diet containing 62 μg/kg body weight SeMet (25 μg/kg bw/day in Se). The administered dose was calculated in accordance with the current dietary reference intakes for human adults. All groups, except for the N group received ethanol (30%, *v*/*v*) by gavage (10 mL/kg bw/day). The body weights of all animals were recorded weekly prior to intragastric administration. At the end, the mice were sacrificed after 12 h of fasting, and the plasma and livers were collected from each mouse and weighed.

### 2.8. Blood Parameters and Enzyme Activities

For biochemical analyses, the blood samples were centrifuged at 3000 rpm and 4 °C for 10 min. Serum concentrations of aspartate transaminase (AST), alanine transaminase (ALT), lactate dehydrogenase (LDH), serum total cholesterol (CHO) and serum total triglycerides (TG) were monitored by a 3100 automatic biochemistry analyzer (Hitachi Ltd., Tokyo, Japan). Insulin levels were detected by an enzyme-linked immunosorbent assay kit (Mercodia, Sweden) [29].

A liver homogenate was prepared in nine volumes of ice-cold physiological saline, and the supernatant was assayed for enzyme activities. According to Da Silva et al. [30], the supernatant of the liver homogenate was prepared for antioxidant enzyme activity under the manufacturer’s instructions of the diagnostic kits from the Nanjing Jiancheng Bioengineering Institute (China), such as glutathione peroxidase (GSH-Px) activity, catalase (CAT) activity, glutathione (GSH) and malondialdehyde (MDA) contents. The activity of cytochrome P4502E1 (CYP2E1) was measured by a CYP2E1 assay kit (Jiangsu Jingmei Biotechnology Co., Ltd., Yancheng, China) according to the instructions.

### 2.9. Statistical Analysis

Statistical analyses were conducted using SPSS 2.0 (IBM Corporation, NY, USA) version 2.0. One-way analysis of variance (ANOVA) was used to determine statistical differences among the treatment groups, and means were separated using Tukey’s multiple comparison test. The significance level was set at α = 0.05. All data were presented as mean ± standard error of the mean (SEM).

## 3. Results

### 3.1. SePP Reversed the Alcohol-Induced Cell Viability Reduction and Cell Death

**Figure 1:**
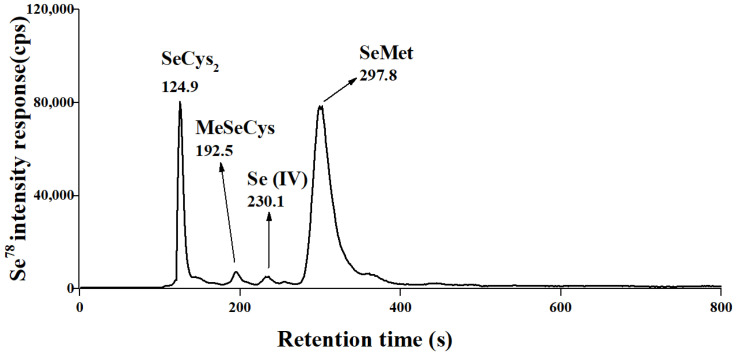
HPLC-ICP–MS chromatogram of Se species in SePP, Peak 1 = Selenocystine (SeCys_2_, 124.9 s); Peak 2 = Se-methylselenocysteine (MeSeCys, 192.5 s); Peak 3 = Se (IV) (230.1 s); Peak 4 = SeMet (297.8 s).

Nutritional composition analysis of a 100 g freeze-dried SePP powder sample showed that it contained 90.91 ± 1.15 g proteins, 2.42 ± 0.06 g fats, 0.25 ± 0.05 g crude fibers, 1.22 ± 0.05 g ashs, 4.23 ± 0.21 g water, and 0.97 ± 0.01 mg Se detected by ICP-MS. The concentration of SeMet analyzed by HPLC-ICP-MS in the freeze-dried SePP was 1.55 ± 0.01 mg (0.62 mg in Se, 85.10%). The other Se species identified in SePP were SeCys_2_ (12.28%), MeSeCys (1.10%) and Se (IV) (1.52%) (Figure 1).

**Figure 2:**
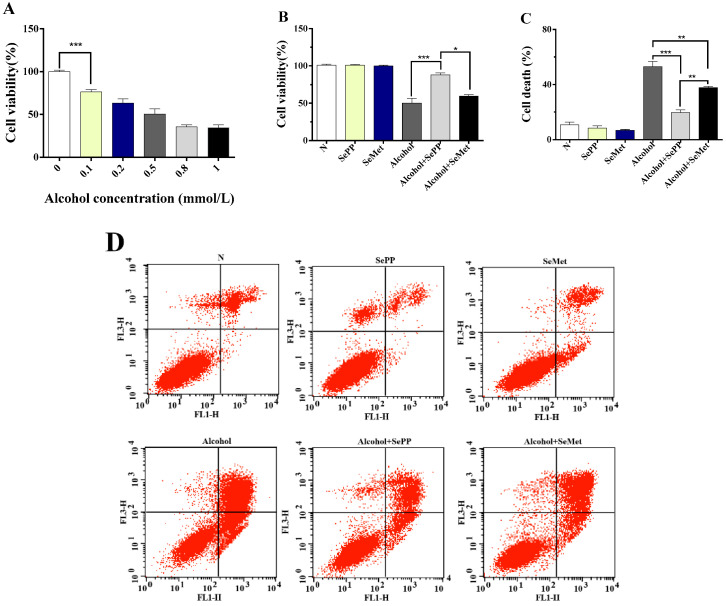
Preventive effects of SePP and SeMet on ALD in a cell culture model. AML-12 cells were treated with different concentrations of alcohol (**A**), effects of alcohol and selenium compounds on cell viability (**B**), and cell death (**C**), flow cytometry scatter plot of cell death (**D**), Data were expressed as mean ± SEM (*n* = 6), * *p* < 0.05, ** *p* < 0.01, *** *p* < 0.001 (the same below unless otherwise notified).

Reduction in cell viability of AML-12 cells as a result of alcohol consumption was dependent on alcohol concentration (Figure 2A). The 24 h exposure to 500 μM alcohol reduced the viability of AML-12 cells to about 50% that in cells without exposure alcoholic exposure. In contrast, cell viabilities of 10 μM SePP or SeMet treated cells increased to 88% (*p* < 0.001) and 59% (*p* > 0.05), respectively (Figure 2B). Moreover, 24-h exposure to either 10 μM SePP or SeMet made no difference in cell viability from the control group.

Cell death measured by flow cytometry was significantly lower in AML-12 cells treated with 10 µM SePP 20% (*p* < 0.001) and SeMet 38% (*p* < 0.01) compared to cells treated with alcohol (53%) (Figure 2C,D).

These results suggest that both SePP and SeMet can protect AML-12 cells from alcohol-induced injuries. SePP showed significantly better protection than SeMet.

### 3.2. SePP Suppressed Alcohol-Induced Increase in Oxidative Stress In Vitro

**Figure 3:**
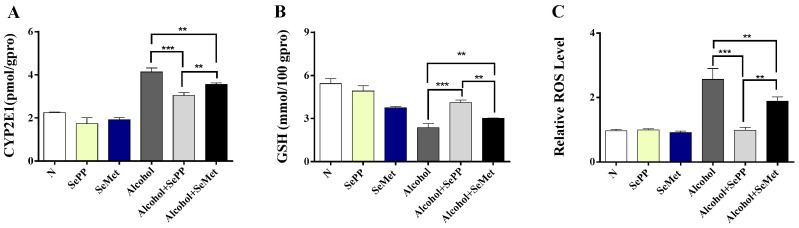

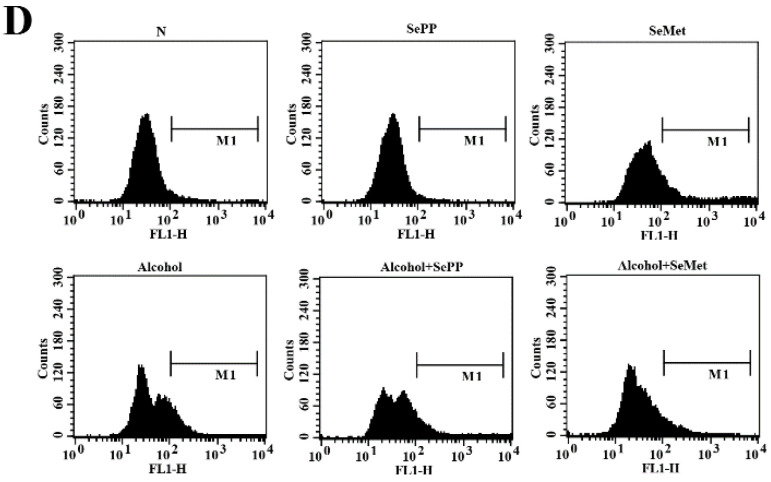
Effects of SePP and SeMet on alcohol-induced increase in oxidative stress in a cell culture model. Effects of alcohol and selenium compounds on CYP2E1 activity (**A**), mitochondrial GSH content (**B**), and relative ROS level (**C**), flow cytometry peak diagram of ROS level (**D**). ** *p* < 0.01, *** *p* < 0.001.

Alcohol induced the activation of CYP2E1 (1.84 folds), resulting of ROS (2.65 folds) production and mitochondrial GSH content depletion (43%) compared with the control group (Figure 3A–D). The productions of CYP2E1 and ROS were markedly suppressed by both treatments with 10 μM SePP (both *p* < 0.001) and 10 μM SeMet (both *p* < 0.01). The Se-dependent treatments also significantly increased the production of mitochondrial GSH (10 µM SePP: *p* < 0.001, SeMet: *p* < 0.01) (Figure 3A–D).

**Figure 4:**
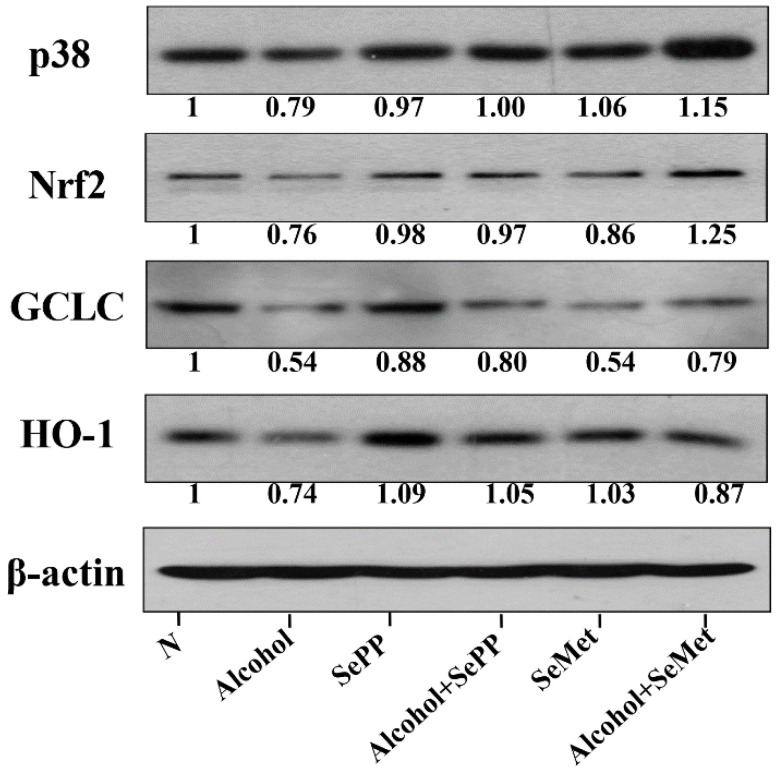
Western blotting of expressions of *p38*, *Nrf-2*, *GCLC*, *HO-1*.

Western blotting was used to detect the expressions of *Nrf-2* and its two transcriptional targets *HO-1* and *GCLC* in cells. After alcohol treatment, the expressions of *Nrf-2* significantly decreased and the same as its two transcriptional targets, *HO-1* and *GCLC* (Figure 4). In comparison, SePP or SeMet treatment resulted in a significant recovery of *Nrf-2*, *HO-1* and *GCLC*.

We further studied the possible mechanism that selenium compounds induced *Nrf-2* activation. The expression levels of the three key MAPK members of phosphorylation state (*ERK1/2, p38*, and *JNK1/2*) were further measured by Western blotting. Results showed that Se-containing compounds such as SePP and SeMet increased the expression of *p38*, but had no significant effect on *JNK1/2* or *ERK1/2*.

### 3.3. SePP Reversed Alcohol-Induced Enhancement of Lipogenesis and Serum Insulin Levels in Mice

**Figure 5:**
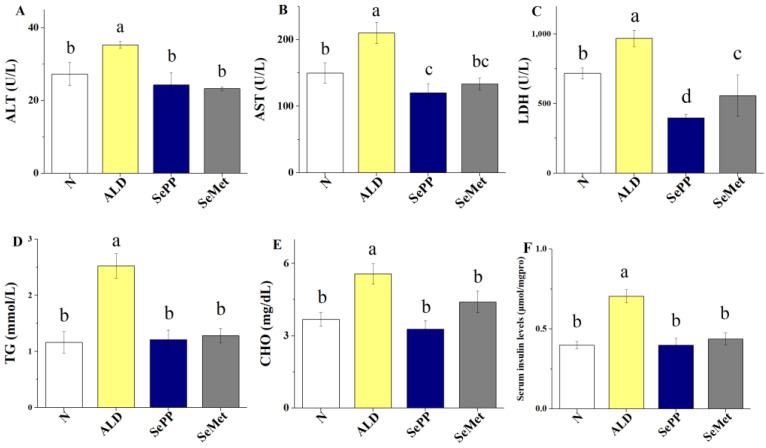
Hepatic lipid and serum insulin levels in mice. Levels of ALT (**A**), AST (**B**), LDH (**C**), TG (**D**), CHO (**E**) and serum insulin levels (**F**). Data are expressed as mean ± SEM (*n* = 9). Different letters denote significant differences between groups (*p* < 0.05) (the same in Figure 6).

To further illustrate the protective effect of Se compounds on ALD, we quantified the levels of ALT and AST, two key biochemical indicators of liver injury in mice. Clearly, alcohol exposure (ALD group) caused a significant increase in ALT and AST levels (Figure 5A,B). Supplementation with SePP or SeMet significantly decreased the levels of ALT, AST, LDH, TG and CHO (*p* < 0.05) in ALD mice (Figure 5A–E). Compared with the SeMet treatment, the levels of AST, LDH, TG and CHO in the SePP group were lower. The levels of serum insulin in ALD mice were markedly higher than in the control group (*p* < 0.05; Figure 5E). The serum insulin levels of the SePP and SeMet groups were similar to those of the control group. These results imply that treatment of the diets with SeMet and especially SePP alleviated ALD.

### 3.4. SePP Suppressed the Alcohol-Induced Increase in Oxidative Stress in Mice

**Figure 6:**
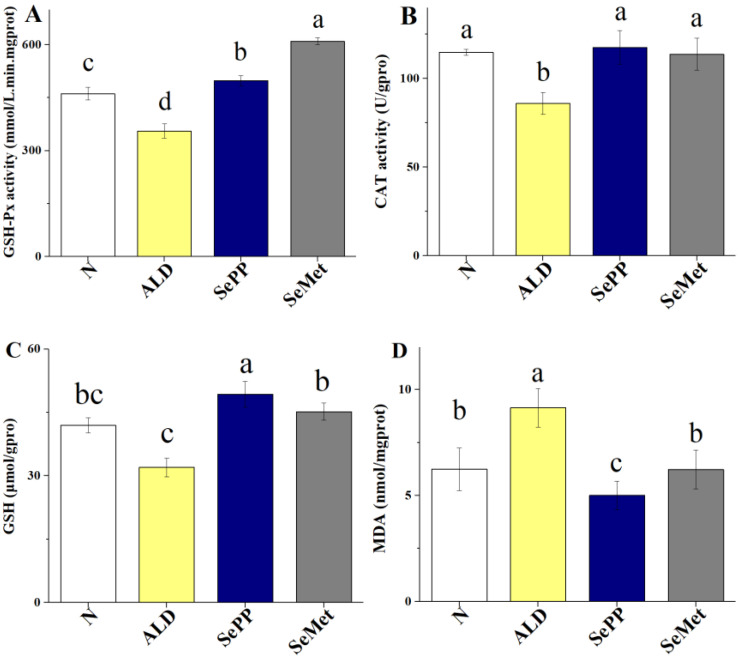
Oxidative damages in mice. Levels of serum GSH-Px activity (**A**), liver CAT activity (**B**), liver GSH depletion (**C**) and MDA generation (**D**) are shown. Different letters denote significant differences between groups (*p* < 0.05).

Oxidative damages in mice represented by serum GSH-Px activity, liver CAT activity, liver GSH depletion, and MDA generation are shown in Figure 6. Results show that levels of serum GSH-Px activity and liver GSH in both the SePP and SeMet groups are significantly higher and MDA levels are lower than in the ALD group (*p* < 0.05) (Figure 6A,C,D), suggesting that the Se-containing interventions can assuage alcohol-induced oxidative stress. CAT activity insignificantly increased in the SeMet group compared to the ALD group (Figure 6B).

These results indicate that the alcohol-induced increase in oxidative stress and serum insulin levels can be suppressed by SePP (50 μg/kg bw/day by Se) in the form of SeMet.

## 4. Discussion

Se has attracted scientific attention for its beneficial effects against antioxidant activity-related diseases, including liver diseases. Most Se-related disease chemoprevention studies utilize commercial reagents, such as sodium selenite, methylseleninic acid, and selenocysteine [31]. However, the biological functions of the active components in Se-enriched foods remain poorly understood [32].

Peanuts are rich in proteins, but research shows that the Se content in peanuts is generally far lower than most natural plant-derived Se-containing foods (0.15 vs. 1–4 μg/g) [33]. After Se enrichment, Se content in the peanuts reached 4.48 μg/g. The Se concentration in freeze-dried SePP is 9.71 μg/g, which falls within the range expected for Se-enriched foods and makes it appropriate for use as a daily Se supplement [34]. SeMet, the major selenocompound in cereal grains, grassl and legumes, and selenized yeast [35,36], is considered as an enrichment substance owing to its better absorption and higher safety than inorganic Se compounds, such as sodium selenate, and sodium selenite [37]. Reportedly, 66% and 22% of the Se in Se-yeast and cashew nuts, respectively, exist in the form of SeMet [32]. The dominant Se species in SePP is SeMet (about 15.50 μg/g), accounting for 85% of the organic Se. Se is principally supplemented as SeMet, as it is the predominant Se species found naturally in foods and is most easily absorbed by the human body [15]. Hence, comparisons in the following analyses on SePP will be made with SeMet.

Our results showed that 24 h treatment with concentrations up to 10 μM of SePP or SeMet caused no toxicity in AML-12 and can reverse the reduction in alcohol-induced cell viability and reduce cell death. These results are in line with a study that 10 μM selenium MeSeCys can reverse the reduction in the viability of HepG2 cells caused by tert-butyl hydroperoxide [38]. Besides, SePP showed a significantly better protective effect than SeMet. These results imply that SePP may play a more important role than low-molecular-weight Se-compounds (e.g., SeMet) and has a certain mitigation effect on alcohol cytotoxicity. This may be attributed to the combined effect of the Se-compound and its associated proteins, or the multiple factors of Se-containing proteins [39].

Se compounds can affect cell growth, probably because it impacts DNA stability, cell proliferation and apoptotic cell death by regulating oxidative stress and immune system [40]. The development of alcoholic liver injury is closely related to the accumulation of ROS. The decrease in some antioxidant active substances (e.g., GSH) negatively impacts on the liver [41]. SeMet can be used as an antioxidant to protect cells against oxidants, such as peroxynitrite [42]. Our study revealed that alcohol depleted GSH levels, and generated ROS. Both SePP and SeMet can suppress oxidative stress. Reportedly, the CYP2E1 system is a potential source of ROS in ALD hepatocytes [2,43,44]. This is consistent with the results of the current study that the activity of CYP2E1 was enhanced by alcohol consumption [45], and the resultant cytotoxicity can be reduced by SePP and SeMet.

Research results shows that overexpression of CYP2E1 can activate *Nrf-2*, which in turn plays a protective role in alcohol-induced liver injury [1,46]. In this study, Se-containing compounds, such as SePP or SeMet, can activate *Nrf-2*, which increases the expression of *Nrf-2* and its two transcription targets, *HO-1* and GCLC. To further explore the effect of SePP or SeMet on activating the upstream mediator of *Nrf-2*, we studied the effects on the three key MAPK members *ERK1/2*, *p38* and *JNK1/2*. Results demonstrated that SePP or SeMet only promoted the expression of p38, but had no positive effect on *ERK1/2* or *JNK1/2* [28,47]. This is in line with reports that the activation of *p38* downstream mediators of *CYP2E1-LPS* increased the likelihood of hepatotoxicity [45,48]. Moreover, relevant related reports show that SeMet can inhibit oxidative stress and relieve myocardial inflammation by regulating *p38* MAPK [49].

The activities of AST, ALT, LDH, and the levels of lipids (e.g., CHO and TG) in the serum can be used as indicators of early liver damage [50,51]. Reportedly, the active substances can relieve liver damage by inhibiting oxidative stress and improving insulin resistance [52]. Here, we also evaluated the effects of Se compounds on the serum levels of LDH, CHO, TG, and insulin as well as the indicators of oxidative damage (e.g., GSH-Px activity, CAT activity, GSH, and MDA content). Results showed that Se compounds such as SePP and SeMet significantly reduced lipogenesis and suppressed alcohol-induced increase in oxidative stress. These results are consistent with another report that the Se-containing compounds extracted from *Schisandra* can significantly improve the activities of antioxidant enzyme (e.g., GSH-Px, CAT), and cell survival rate [53]. Selenium polysaccharides extracted from *Glycyrrhizae* could significantly inhibit the serum ALT and AST levels in mice caused by the release of free radicals [53]. Moreover, most Se-proteins exhibit antioxidant activities, which are correlated with lipid oxidation and the profile of antioxidative enzymes, such as GSH-Px, selenoproteins, glutathione reductase (GR), and SOD [54,55,56]. Reportely, Se mediates several insulin-regulated events and pro-insulin-like actions, including the effects on sugar metabolism and lipid metabolism [45,57]. In addition, alcoholic patients and liver disease patients always have low serum Se levels, which implies that Se compound supplementation will work for ALD [57,58]. Therefore, supplementation with SePP and SeMet reduced the level of oxidative stress, and regulated lipid metabolism, therefore reducing the effects of alcohol-induced liver damage in mice.

Se compounds, including inorganic Se, and organic Se (e.g., SeMet, SeCys) impact the composition of intestinal microbes [59]. Supplementation with a certain amount of selenium can significantly increase the abundance of intestinal lactic acid in mice. Se-enriched *lentinan* can slow down the pancreatic damage in mice by regulating the composition of intestinal flora [59]. Se-enriched foods are believed to contribute to the abundance of probiotics [60,61]. Dietary Se-enriched green tea and Se-enriched *Bifidobacterium longum* DD98 are beneficial to the growth and reproduction of intestinal lactic acid bacteria [60]. Other forms of organic Se have similar results. Chronic alcohol consumption results in an imbalance of intestinal microbiota by changing the permeability of intestinal mucosa [62]. Cellular metabolites such as endotoxin after entering the blood will activate cells to release free radicals and cytokines by binding to specific receptors of liver cells, which in turn promote the release of abundant inflammatory mediators and cause damage to liver cells [63,64]. In the future, whether SePP suppresses oxidative stress by modulating the composition of gut microbiota deserves to be studied.

## 5. Conclusions

A certain concentration of SeMet-dominated SePP alleviated alcohol-induced AML-12 cytotoxicity, and one of the important mechanisms was the suppression of oxidative stress. Additionally, *p38*-dependent mechanisms contribute to SePP-induced *Nrf-2* activation. Supplementation with SePP and SeMet reduced the level of oxidative stress, and regulated lipid metabolism to reduce the effects of alcohol-induced liver damage in mice. Our findings indicate that SeMet-dominated SePP has potential applications as a nutraceutical for ALD prevention.

In the future, whether SePP suppresses oxidative stress by modulating the composition of gut microbiota deserves to be studied. On the other hand, further research can focus on the bioavailability evaluation to clarify whether there are interferences among SeMet, other Se compounds and other substances in absorption by SePP. These studies will provide an important basis for the deep processing of Se-enriched agricultural products.

## Data Availability

The datasets generated for this study are available on request to the corresponding author.
